# Bushen Huoxue recipe attenuates early pregnancy loss via activating endometrial COX2-PGE2 angiogenic signaling in mice

**DOI:** 10.1186/s12906-021-03201-9

**Published:** 2021-01-14

**Authors:** Yufan Song, Fanru Zhou, Xiujuan Tan, Xia Liu, Jiahui Ding, Chu Zhang, Fan Li, Wenxin Zhu, Wenwen Ma, Runan Hu, Mingmin Zhang

**Affiliations:** 1 Institute of Integrated Traditional Chinese and Western Medicine, Tongji Hospital, Tongji Medical College, Huazhong University of Science and Technology, Wuhan, 430030 People’s Republic of China; 2 Department of Obstetrics and Gynecology, School of Medicine, Wayne State University, Detroit, MI USA

**Keywords:** Traditional Chinese medicine, Bushen Huoxue recipe, Early pregnancy loss, Hormone balance, Endometrial angiogenesis

## Abstract

**Background:**

During the fresh cycles of in vitro fertilization and embryo transfer, a disturbance in the reproductive endocrine environment following controlled ovarian hyperstimulation (COH) is closely related to compromised endometrial receptivity. This is a major disadvantage for women during pregnancy. Based on the theory of traditional Chinese medicine, Bushen Huoxue recipe (BSHXR) has been indicated to facilitate embryo implantation.

**Methods:**

The COH model (Kunming breed) was induced by injecting mice with pregnant mare serum gonadotrophin (0.4 IU/g) and human chorionic gonadotropin (1 IU/g), followed by treatment with BSHXR at three different concentrations (5.7, 11.4, and 22.8 g/kg), Bushen recipe (BSR) (5.7 g/kg), and Huoxue recipe (HXR) (5.7 g/kg). After successful mating, the pregnancy rate and implantation sites were examined on embryo day 8 (ED8), and the weight ratio of endometrium was calculated on ED4 midnight. Serum estrogen, progesterone, and endometrial PGE2 levels were measured using enzyme-linked immunosorbent assay. The endometrial microvasculature was evaluated using CD31 immunostaining. The protein and mRNA levels of the angiogenic factors in the endometrium were evaluated using western blot, immunohistochemistry, and polymerase chain reaction.

**Results:**

In the COH group, the pregnancy rate and implantation sites were significantly decreased, and abnormal serum hormone levels and impaired endometrial vascular development were observed. After BSHXR treatment, the supraphysiological serum progesterone level in COH mice was restored to normalcy. Moreover, the abnormal expression of the endometrial pro-angiogenic factors, including HIF1α, COX2-PGE2 pathway, and the down-stream factors, namely, MMP2, MMP9, TIMP2, and FGF2 after subjecting mice to COH was significantly improved after BSHXR treatment.

**Conclusion:**

BSHXR could improve embryo implantation by regulating hormonal balance and modulating endometrial angiogenesis in mice, without inducing any side effects in normal pregnancy.

**Supplementary Information:**

The online version contains supplementary material available at 10.1186/s12906-021-03201-9.

## Background

Infertility is a global issue that impacts 10% of women who are of childbearing age; however, this incidence can be as high as 30% in developing countries [1, 2]. Owing to the rapid development of assisted reproduction technology, an increasing number of infertile couples choose in vitro fertilization and embryo transfer (IVF-ET) methods to achieve pregnancy. Nevertheless, the implantation rate is relatively low, and several couples undergo recurrent implantation failure [3, 4]. Controlled ovarian hyperstimulation (COH) is a necessary strategy to obtain considerable numbers of high-quality oocytes and embryos in IVF-ET cycles. However, the exposure to supraphysiological levels of sex hormones resulting from ovarian hyperstimulation may interfere with the process of embryo implantation, which is an intractable problem in the field of IVF-ET [5].

During pregnancy, the endometrium is actively, rather than passively, involved in embryo implantation, thereby creating an effective maternal-fetal interaction [6]. Successful embryo implantation requires the induction of a highly programmed sequence of cellular events. In mice, embryos enter the uterine cavity on embryo day (ED) 4 morning, and continue with positioning and communicating with the epithelium until the evening of ED4 [7]. Upon adhering to the luminal epithelium, the trophectoderm starts to invade on ED4 midnight and is then buried into the endometrium on the evening of ED5, which indicates the completion of implantation [7]. All of these steps are critically governed by steroid hormones [8]. Accumulating evidence reveals that high levels or a rapid elevation in the levels of estrogen, progesterone, or human chorionic gonadotropin (HCG) could be associated with suboptimal and asynchronous endometrial development, resulting in implantation failure [9].

Attempts at focusing on enhancing endometrial receptivity have been carried out in recent years, including endometrial injury and the use of drugs, such as aspirin, heparin and sildenafil, which may improve the microcirculation and trophoblast invasion during pregnancy [10, 11]. However, additional evidence is required to confirm the efficacy and mechanisms of these processes [12–14]. Further, frozen embryo transfer (FET) has been more frequently adopted to prepare a “healthy” endometrium to avoid the side effects in fresh cycles. However, results on the long-term safety of FET appear inconclusive. A recent meta-analysis reveals that the pregnant women or neonates undergoing FET procedure could pose a higher risk of maternal hypertensive disorders, large for gestational age, or high birth weight [15]. Given these findings, seeking better methods to promote endometrial receptivity in fresh cycles is worth exploring.

Traditional Chinese medicine (TCM) has gained wide acceptance owing to its advantages of multi-factorial and multi-target actions. A study with 1231 IVF patients reports a greater odds of live birth after whole-systems TCM treatment [16]. Bushen Huoxue recipe (BSHXR) is utilized by a large number of individuals in treating gynecological diseases [17]. Studies report that Bushen Huoxue formula can improve endometrial development [18], modulate the immune microenvironment in the endometrium [19], and increase the high-quality embryo rate [20], thereby facilitating the pregnancy outcome. A recent research indicates that Bushen Tiaoxue granules (a decoction based on Bushen Huoxue) could significantly promote the endometrial receptivity via rescuing the impaired expression of vascular endothelial growth factor (VEGF) A, LPA3, p53, and LIF in the endometrium of rats that underwent COH [21]. Nevertheless, more evidence is needed to elucidate the mechanism of Bushen Huoxue in promoting pregnancy.

In our previous studies, we established a mouse model using ovarian hyperstimulation to simulate disturbed steroid hormone levels and abnormal endometrial development during fresh embryo transfer. A higher post-implantation loss was observed in mice that underwent COH, and BSHXR prevented this loss by facilitating angiogenesis via fibroblast growth factor 2 (FGF2) in the endometrium from ED5 to ED8 [22, 23]. In this study, we aimed to further explore the mechanism of BSHXR in regulating endometrial angiogenesis on ED4 midnight, a time point critical for the initiation of implantation. Together, this work could provide a scientific basis for the clinical use of BSHXR during the peri-implantation period in IVF-ET, ensuring successful embryo implantation in infertile patients.

## Methods

### Medicine preparation

Medicine granules were produced by HuaRun SanJiu Medical and Pharmaceutical Co., Ltd. (Shenzhen, China). BSHXR formulation consists of six herbs (Table 1). High performance liquid chromatography of BSHXR has been described in our previous study [22]. Three different doses of BSHXR were prepared. The concentrations of 5.7, 11.4, and 22.8 g/kg of the preparations were designated as BSHXR-L (low dose), BSHXR-M (middle dose), and BSHXR-H (high dose), respectively. Furthermore, BSR (Tu si zi, Sang ji sheng and Xu duan) and HXR (Huang qi, Dang gui and Chuan xiong) were separately prepared according to the dosage of BSHXR-M, both at a concentration of 5.7 g/kg. Calculating details have been demonstrated previously [22]. Before use, the granules were added to boiling water and stirred until they were completely dissolved.

**Table 1:** Components of BSHXR

Chinese name	Latin name	Family	Voucher number	Plant Part	Weight (g)
Huang qi	*Astragali radix*	*Astragalus aaronii* (Eig) Zohary	J4013–6115	Root	14
Dang gui	*Angelicae sinensis radix*	*Angelica sinensis* (Oliv.) Diels	J4012–6111	Root	12
Chuan xiong	*Ligustici Chuanxiong Rhizoma*	*Ligusticum acuminatum* Franch.	J4013–6128	Root	12
Tu si zi	*Cuscutae semen*	*Cuscuta chinensis* Lam.	J4013–6119	Seed	14
Sang ji sheng	*Taxilli Herba*	*Loranthus acaciae* Zucc.	J4013–6131	Stem and branch	12
Xu duan	*Dipsaci Radix*	*Dipsacus acaulis* (A.Rich.) Napper	J4013–6132	Root	12

### Animal operating procedure

Eight-week-old female (*n* = 880, weight: 25–30 g) and ten-week old male (*n* = 100, weight: 35–40 g) Kunming mice were used in this study. The mice were procured from the Laboratory Animal Center of Tongji Medical College, Huazhong University of Science and Technology. They were raised in a specific pathogen free environment (20 ± 2 °C, 60 ± 5% humidity, 12 h: 12 h light/dark cycle) and given free access to water and food (5 female mice/cage, 1 male mouse/cage). This study was approved by the Ethics Committee on the Animal Experimentation of Tongji Medical College, Huazhong University of Science and Technology, Wuhan, China (Project No: S-2012-352).

After 1 week of acclimatization, the female mice were randomly assigned into eight groups (*n* = 110/group) as follows: control group, control + BSHXR group, model group, BSHXR-L group, BSHXR-M group, BSHXR-H group, BSR group, and HXR group. The control + BSHXR group comprised normal mice that were administrated BSHXR-M. Vaginal smears were checked daily in the morning to observe the estrous cycle. Only mice with two successive regular estrous cycles were included in subsequent studies. Next, a modified COH model was established based on the protocols reported in our previous study [23]. Specifically, female mice in the model group and in all treatment groups were intraperitoneally injected with 0.4 IU/g pregnant mare serum gonadotrophin (PMSG) (Hangzhou Animal Medicine Factory, China) at diestrus. Then, distilled water (0.01 mL/g body weight) was administered to the mice in the control, control + BSHXR, and model groups. A similar volume of 0.01 mL/g of BSHXR (three doses included), BSR, or HXR was administered daily to mice in the treatment groups by oral gavage until the day of sacrifice. After 48 h of PMSG injection, an injection of 1 IU/g HCG (Lizhu Pharmaceutical Factory, China) was administered at estrus. An injection of 95% saline was simultaneously administered to the control and control + BSHXR groups. Then, the female mice were mated with male mice overnight in the independent cages. The following morning, a vaginal plug was considered as an indicator of successful copulation, and this day was regarded as ED1.

On Day 1 after PMSG (24 h after PMSG), ED1 (2 p.m.), ED4 midnight (11 p.m.), and ED8 (2 p.m.), sodium pentobarbital was injected intraperitoneally (70 mg/kg, Goodbio Technology Co., China). Necessary measures were adopted to minimize pain and suffering to the animals during experiments. After the mice were deeply sedated, which was determined based on the disappearance of corneal reflexes and the sensation of pain, we obtained blood samples from the orbital vein. Cervical dislocation was performed and the mice were sacrificed. The blood samples were centrifugated (3000 rpm × 15 min) and the serum was collected to detect the levels of steroid hormones. The pregnancy rate (number of pregnant mice/number of vaginal positive mice) and the number of implantation sites (total number of implantation sites/total number of pregnant mice) on ED8 were recorded. On ED4 midnight, we rinsed the uterine cavity with 95% saline to remove the embryos. Then, the endometrium was scraped out using a bent needle and frozen promptly for RNA and protein extraction and prostaglandin E 2 (PGE2) measurement. The procedure of this experiment is shown in Additional file 2.

### Enzyme-linked immune sorbent assay (ELISA)

Estrogen (R&D, USA), progesterone (Demeditec, Germany), and PGE2 (R&D, USA) levels were determined using an ELISA kit according to the manufacturer’s instructions.

### Quantitative real-time PCR

Trizol (TaKaRa, Japan) was used to extract the total RNA from the endometrial tissue. After the determination of RNA concentration, cDNA was synthesized using a reverse transcription reagent (TaKaRa, Japan). Then, the SYBR Green qPCR kit (TaKaRa, Japan) was used for Quantitative Real-Time PCR (qRT-PCR) (Applied Biosystems, USA). Specific primer sequences are enumerated in Additional file 3. The 2^-∆∆CT^ method was used to determine the relative expression level of mRNA.

### Western blotting

The endometrial tissue was mixed with lysis buffer and the protease inhibitor. Then, the samples were homogenized and centrifuged (12,000 rpm × 10 min), and the supernatants were collected. After determining the protein concentration, the samples were subjected to sodium dodecyl sulfate-polyacrylamide gel electrophoresis (SDS PAGE) and transferred to PVDF membranes. The membranes were blocked with 5% nonfat milk for 1 h at room temperature and then incubated with the primary antibody overnight at 4 °C. The antibodies included anti-estrogen receptor α (ERα, Santa Cruz Biotechnology, USA), anti-progesterone receptor A (PRA, Proteintech, China), anti-hypoxia inducible factor 1α (HIF1α, R&D, USA), anti-VEGFA (Proteintech, China), anti-angiopoietin 2 (ANGPT2, Abcam, USA), anti-cyclooxygenase 2 (COX2, CST, USA), anti-prostaglandin E receptor 2 (EP2 receptor, Abcam, USA), anti-matrix metallopeptidase 2 (MMP2, Abcam, USA), anti-MMP9 (Abclone, China), anti-tissue inhibitor of metalloproteinase 2 (TIMP2, R&D, USA), anti-FGF2 (Santa Cruz Biotechnology, USA), and anti-β-actin (Proteintech, China). Next, the PVDF membranes were incubated with the fluorescent secondary antibodies (CST, USA) at room temperature for 1 h. Lastly, the protein bands were scanned using the Odyssey Infrared Imaging System (Li-Cor Biosciences, USA).

### Immunohistochemistry

Isolated uteri were fixed in paraformaldehyde and embedded in paraffin. After dewaxing with xylene and subjecting it to a gradient concentration of alcohol, the antigen was retrieved. The tissue sections were blocked with goat serum for 20 min at room temperature. Then, the sections were incubated with primary antibodies (anti-CD31, Abcam, USA; anti-HIF1α, R&D, USA; anti-COX2, CST, USA; anti-EP2 receptor, Abcam, USA; anti-MMP2, Abcam, USA; anti-MMP9, Abclone, China) for 1 h at room temperature, followed by incubation with the secondary antibody for 1 h at room temperature. After washing, the sections were developed using 3,3′-diaminobenzidine and stained using hematoxylin for about 3 min. Lastly, the slices were processed by dehydration, hyalinization, and sealing. Images were scanned using a NanoZoomer slide scanner (Hamamastu, Japan) and observed using NDP view2 system.

### Statistical analysis

Statistical analysis was conducted using IBM SPSS 20.0. Normally distributed results were expressed using mean ± SD. One-way analysis of variance (ANOVA) was used for the comparison of differences. In detail, the LSD test was used for data with equal variance, while Dunnett’s T3 test was used to process data with unequal variance. If an abnormal distribution was present, the data were indicated as medians (first quartile, third quartile) and analyzed using the nonparametric test. Additionally, the rates were compared using the Chi-square test. A *p*-value < 0.05 was considered to be statistically significant.

## Results

### BSHXR improved embryo implantation and endometrial development

First, we evaluated the efficacy of BSHXR in mice that were subjected to COH. The body weights were recorded from ED1 to ED8 (Additional file 4). On the whole, during the development of pregnancy, there was an upward trend in the weight of the mice from ED1 to ED8. It could also be seen that the weight of the mice in the control + BSHXR group was higher than that of the control group. There was a further increase in the weight of mice that underwent COH. Treatment with BSHXR had no influence on weight. As shown in Table 2, the pregnancy rate remarkably decreased in the model group compared to that in the control group (*P* < 0.01). After treatment, BSHXR-M could attenuate the pregnancy loss (*P* < 0.05). Moreover, no notable differences were found between the control and control + BSHXR groups.

**Table 2:** Effects of BSHXR on pregnancy outcome on ED8

Group	Pregnant Number/Total Number	Pregnancy Rate (%)	Median Number of Implanted Embryos
Control	14/16	87.50	15.5 (14, 17.75)
Control + BSHXR	10/12	83.33	15.0 (14.25, 15.75)
Model	10/27	37.04^**##**^	5.5 (3.25, 8)^**###**^
BSHXR-L (5.7 g/kg)	8/15	53.33	14.5 (4.75, 23.5)
BSHXR-M (11.4 g/kg)	19/28	67.86*	20.0 (11.5, 26)**
BSHXR-H (22.8 g/kg)	10/21	47.62	6.5 (4.25, 16.75)
BSR (5.7 g/kg)	6/14	42.86	22.5 (15.75, 33)
HXR (5.7 g/kg)	7/13	53.85	9.0 (4, 20)

Data with a skewed distribution represent as medians (first quartile, third quartile). ^##^*P* < 0.01, ^###^*P* < 0.001 vs. control Group, *P < 0.05, **P < 0.01 vs. model Group.

**Fig. 1:**
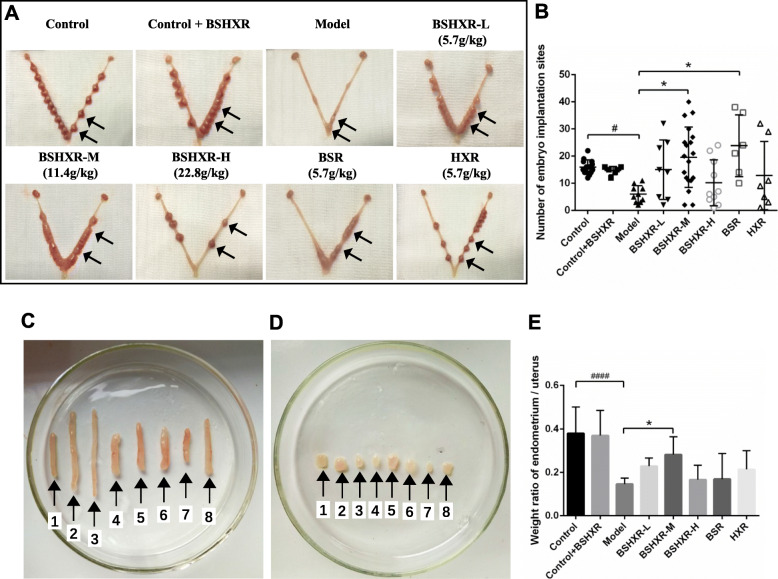
Effects of BSHXR on murine pregnancy and endometrial development. **a** Uterine morphology on ED8. Arrows point the embryo sites. **b** Number of embryo implantation sites. ^#^*P* < 0.05 vs. control group, ^*^P < 0.05 vs. model group. **c** Overall observation of the uterus. **d** Endometrium isolated from the uterus. 1, Control; 2, Control + BSHXR; 3, Model; 4, BSHXR-L; 5, BSHXR-M; 6, BSHXR-H; 7, BSR; 8, HXR. The number of the mice from 1 to 8 were 41–3, 45–2, 48–3, 49–2, 48–4, 49–1, 40–5, 42–2. The first number indicates the number of the cage, and the second number indicates the animal number in each cage. **e** Weight ratio of endometrium to uterus. Data were represented as mean ± SD. ^####^*P* < 0.0001 vs. control group, ^*^P < 0.05 vs. model group

As seen in Fig. 1a, the embryos appear well-developed and show a string-of-beads arrangement along the vertical axis in the control group. While the embryos in the model group appeared tiny and runtish. The treatment profoundly promoted the development and distribution of the embryos, particularly in mice administered BSHXR-L and BSHXR-M. Moreover, the numbers of implantation sites in the control group were relatively uniform. In the control + BSHXR group, BSHXR made the numbers of implantation sites more concentrated. COH significantly decreased the implanted embryos in the model group (*P* < 0.001); however, this phenomenon could be improved to different degrees after treatment, especially in the BSHXR-M and BSR groups (*P* < 0.01) (Fig. 1b).

Next, the uteri and endometria were isolated on ED4 midnight. As shown in Fig. 1c, the uterus in the model group was thin and pale, whereas those in the other groups appeared relatively plump. When isolated from the uteri, the endometria in the control and control + BSHXR groups could be scraped off smoothly. However, the endometrium in the model group was fragmented and scarce. BSHXR and the separated prescriptions could improve the visible morphology of the endometrium, particularly treatment with BSHXR-M (Fig. 1d). Next, the weight ratio of the endometrium: uterus was calculated. The ratio was significantly low in the model group (*P* < 0.0001). After treatment with BSHXR-M, the ratio improved remarkably (*P* < 0.05) (Fig. 1e).

### BSHXR restored the steroid hormone levels and hormone receptors

**Fig. 2:**
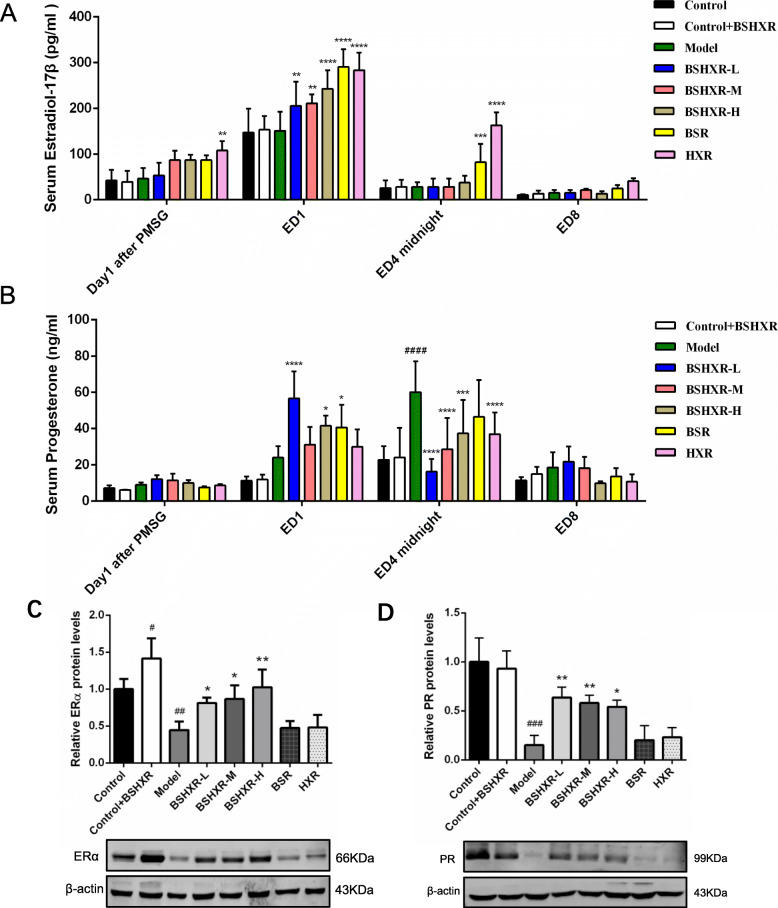
Effects of BSHXR on serum hormones and hormone receptors. **a** Serum estradiol-17β level on Day1 after PMSG, ED1, ED4 midnight, and ED8 (*n* = 6–8). **b** Serum progesterone level on Day1 after PMSG, ED1, ED4 midnight, and ED8 (*n* = 6–8). **c** Endometrial ERα protein level on ED4 midnight (*n* = 3). **d** Endometrial PR protein level on ED4 midnight (*n* = 3). The western blot images in (**c**) and (**d**) were cropped. Data were represented as mean ± SD. β-actin was the reference. ^#^*P* < 0.05, ^##^*P* < 0.01, ^###^*P* < 0.001, ^####^*P* < 0.0001 vs. control group, ^*^P < 0.05, ^**^P < 0.01, ^***^P < 0.001 and ^****^P < 0.0001 vs. model group

To describe the dynamic levels of steroid hormones, serum estrogen (Fig. 2a) and progesterone (Fig. 2b) were measured on Day1 after PMSG, ED1, ED4 midnight, and ED8. Among the control, control + BSHXR, and model groups, there was no notable difference in the estrogen levels. Nevertheless, estrogen was increased in the HXR group on Day1 after PMSG (*P* < 0.01), and in all the treatment groups on ED1 (*P* < 0.0001 or *P* < 0.01). Further, both the BSR and HXR groups showed an increase in estrogen levels on ED4 midnight (*P* < 0.0001 or *P* < 0.001). Progesterone levels were comparable on Day1 after PMSG and ED8. However, there was an increase in progesterone levels in the BSHXR-L, BSHXR-H, and BSR groups on ED1 compared to that in the model group (*P* < 0.0001 or *P* < 0.05). On ED4 midnight, a sharp rise in progesterone was seen in the model group when compared to the control group (*P* < 0.0001). However, this spike was not observed in the BSHXR-L, BSHXR-M, BSHXR-H, and HXR groups (*P* < 0.0001 or *P* < 0.001).

Next, the levels of endometrial ERα and PR protein were determined on ED4 midnight (Fig. 2c and d). Compared to that in the control group, BSHXR further promoted ERα expression in the control + BSHXR group (*P* < 0.05). Although ERα and PR protein levels in the model group were significantly lower than those in the control group (*P* < 0.001 or *P* < 0.01), these levels were found to be increased in the BSHXR-L, BSHXR-M, and BSHXR-H groups (*P* < 0.01 or *P* < 0.05).

### Treatment rescued endometrial angiogenesis

**Fig. 3:**
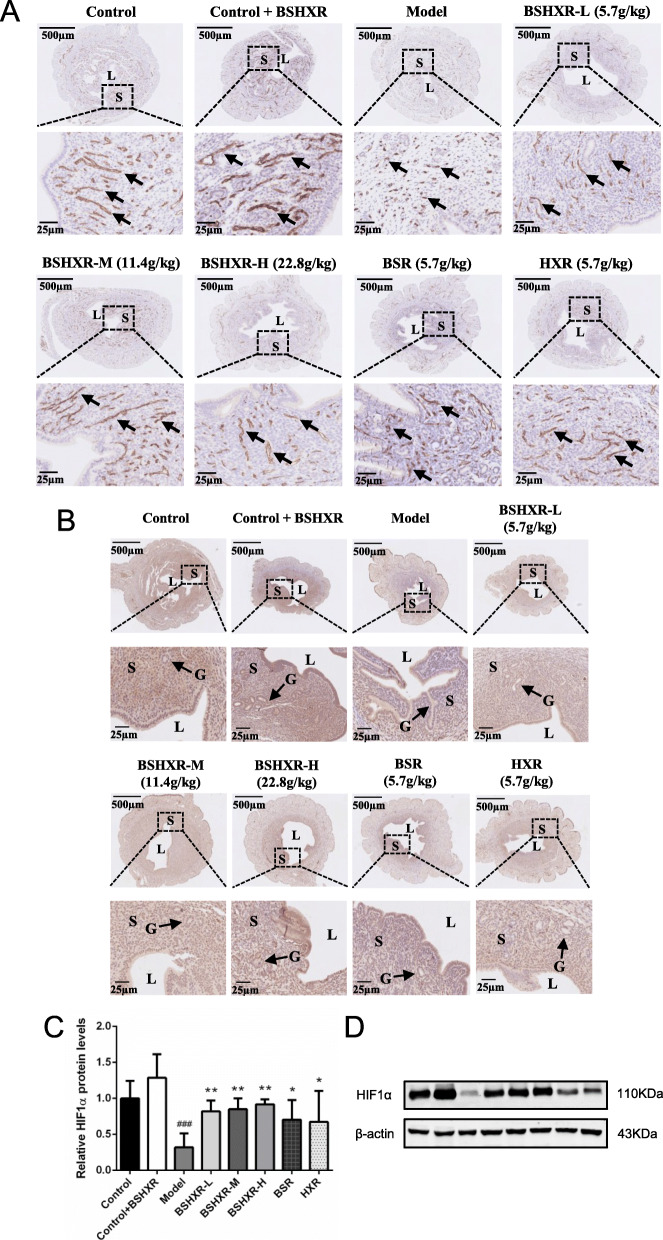
Effects of BSHXR on endometrial angiogenesis. **a** Representative images of CD31 immunohistochemistry staining in the uterus. Scale bar is shown in pictures. Arrow indicates the CD31 staining. L, lumen; S, stroma. **b** Representative images of HIF1α immunohistochemistry staining in the uterus. **c**-**d** Endometrial HIF1α protein level (*n* = 3). The western blot images in (**d**) was cropped. Scale bar is shown in pictures. L, lumen; S, stroma. Arrows indicate the gland (G). Data were represented as mean ± SD. β-actin was the reference. ^###^*P* < 0.001 vs. control group, ^*^P < 0.05, ^**^P < 0.01 vs. model group

To study the importance of angiogenesis in endometrial receptivity, CD31 immunostaining was performed using the endometria obtained on ED4 midnight. As shown in Fig. 3a, CD31 staining was mainly concentrated in the stroma. In the control and control + BSHXR groups, high immunoactivity was detected using CD31 staining, and the microvasculature showed significant elongations and branches. After COH, CD31 staining appeared weak and an immature vascular morphology was observed. On the other hand, microvasculature formation could be significantly improved in the treatment groups .

To explore the mechanism of BSHXR in angiogenesis, the proteins, HIF1α, VEGFA, and ANGPT2 were measured on ED4 midnight. As seen in Fig. 3b, HIF1α is located in the luminal epithelium, glandular epithelium, and stroma. HIF1α expression was notably reduced in the model group compared to that in the control group; however, all the treatment groups could facilitate its expression. The quantification of HIF1α revealed a consistent trend (Fig. 3c and d). However, as for the VEGFA and ANGPT2 proteins, the levels of both were comparable among all eight groups (Additional file 6a and b).

### Regulation of BSHXR on endometrial COX2, EP2 receptor, and PGE2

**Fig. 4:**
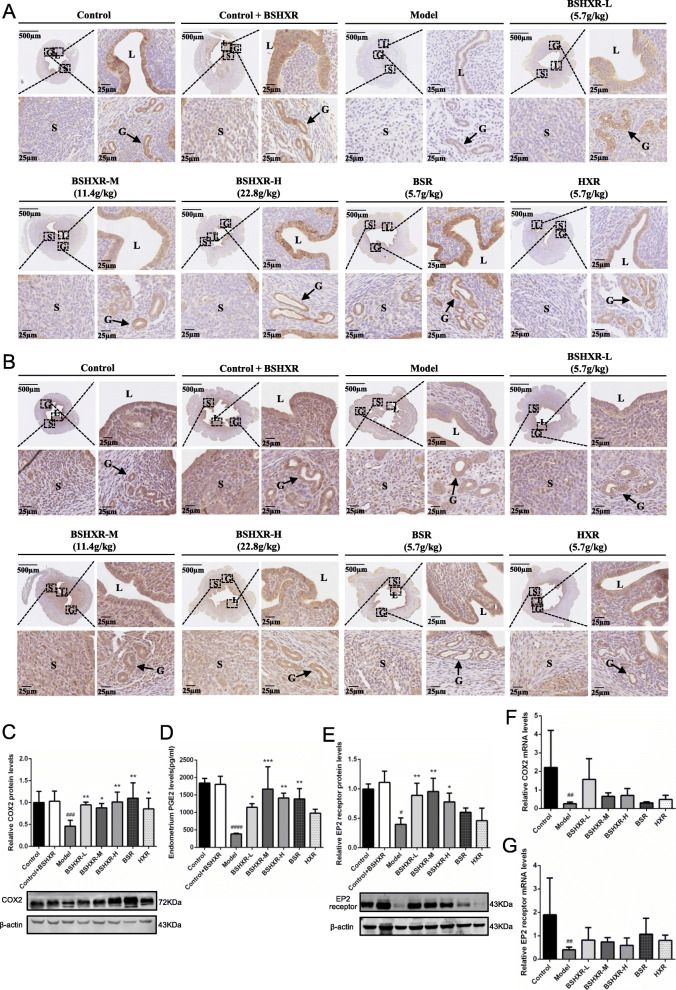
Effects of BSHXR on endometrial COX2, PGE2, and EP2 receptor expression on ED4 midnight. **a** Representative images of COX2 immunohistochemistry staining in the uterus. **b** Representative images of EP2 receptor immunohistochemistry staining in the uterus. **c** COX2 protein level in endometrium (*n* = 3). **d** Endometrial PGE2 level. **e** EP2 receptor protein level in endometrium (*n* = 3). **f** Relative mRNA level of endometrial COX2 (*n* = 5–6). **g** Relative mRNA level of endometrial EP2 receptor (*n* = 5–6). The western blot images in (**c**) and (**e**) were cropped. Scale bar is shown in pictures. L, lumen; S, stroma. Arrows indicate the gland (G). Data were represented as mean ± SD. β-actin was used as the reference. ^##^*P* < 0.01, ^###^*P* < 0.001, ^####^*P* < 0.0001 vs. control group, ^*^P < 0.05, ^**^P < 0.01, ^***^P < 0.001 vs. model group

Proangiogenic COX2-PGE2 signaling was further investigated in samples from ED4 midnight. As shown in Fig. 4a, COX2 protein was mainly expressed in the luminal and glandular epithelium. There was a prominent decrease in the COX2 protein level in the model group (*P* < 0.001); however, after treatment, this trend was reversed (*P* < 0.01 or *P* < 0.05) (Fig. 4c). As a major product of COX2, the PGE2 level in the model group was lower than that in the control group (*P* < 0.0001), while BSHXR and BSR treatment could improve these levels (*P* < 0.001 or *P* < 0.01 or *P* < 0.05) (Fig. 4d). The receptor of PGE2, EP2 receptor, was mainly located in luminal and glandular epithelium, and to some extent in the stroma (Fig. 4b). In COH mice, the immunostaining of EP2 receptor was obviously weakened in the stroma and epithelium. Western blotting also revealed a significant decrease in the EP2 receptor in the model group compared to that in the control group (*P* < 0.05), and treatment with BSHXR could promote it (*P* < 0.01 or *P* < 0.05) (Fig. 4e). In terms of transcription, both COX2 and EP2 receptor mRNA levels in the model group were lower than those in the control group (*P* < 0.01), while the treatment had no effect on them (Fig. 4f and g).

### Regulation of BSHXR on endometrial MMP2, MMP9, TIMP2, and FGF2

**Fig. 5:**
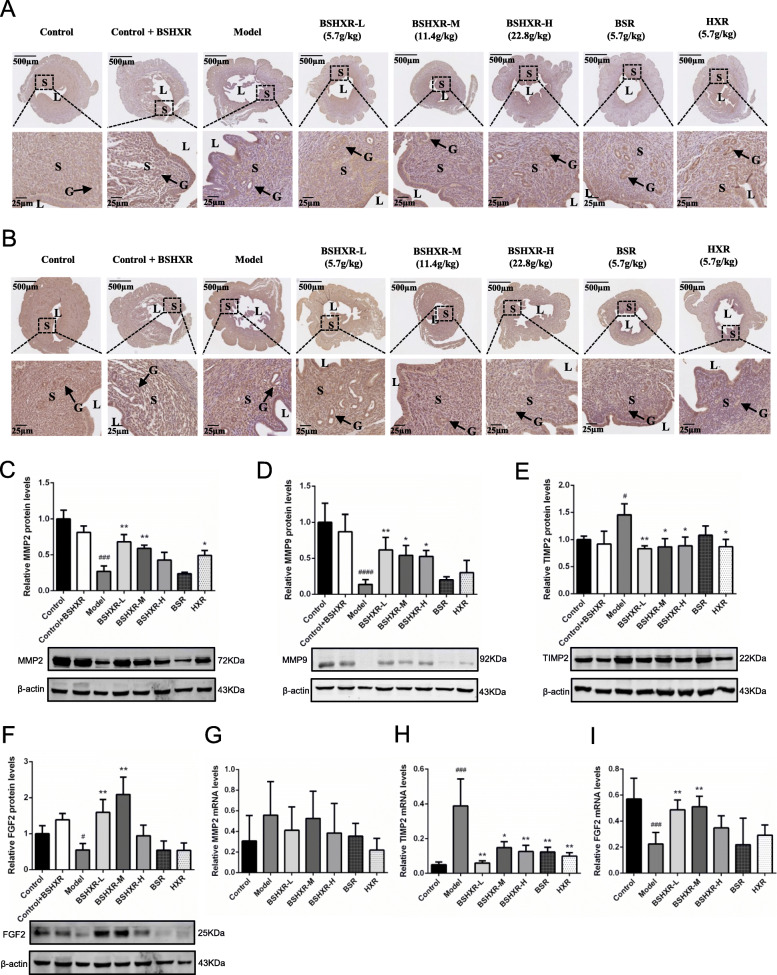
Effects of BSHXR on endometrial MMP2, MMP9, TIMP2, TIMP3, and FGF2 expression on ED4 midnight. **a** Representative images of MMP2 immunohistochemistry staining in the uterus. **b** Representative images of MMP9 immunohistochemistry staining in the uterus. **c** MMP2 protein level in endometrium (*n* = 3). **d** MMP9 protein level in endometrium (*n* = 3). **e** TIMP2 protein level in endometrium (*n* = 3). **f** FGF2 protein level in endometrium (*n* = 3). **g** Relative endometrial MMP2 mRNA level (*n* = 6). **h** Relative endometrial TIMP2 mRNA level (*n* = 5–7). **i** Relative endometrial FGF2 mRNA level (*n* = 5–6). The western blot images in (**c**), (**d**), (**e**), and (**f**) were cropped. Scale bar is shown in pictures. L, lumen; S, stroma. Arrows indicate the gland (G). Data were represented as mean ± SD. β-actin was the reference. ^#^*P* < 0.05, ^###^*P* < 0.001, ^####^*P* < 0.0001 vs. control group, ^*^P < 0.05, ^**^P < 0.01 vs. model group

Next, we examined the downstream factors of COX2-PGE2 signaling on ED4 midnight. As shown in Fig. 5a and b, MMP2 and MMP9 immunoreactivity were evident in the epithelium and stroma. Both MMP2 and MMP9 levels were decreased in the model group, especially in the stroma. Western blotting revealed that MMP2 protein level was decreased in the model group compared to that in the control group (*P* < 0.001). BSHXR-L, BSHXR-M, and HXR elevated the MMP2 level after treatment (*P* < 0.01 or *P* < 0.05) (Fig. 5c). A similar trend in MMP9 protein level was found in the model group (*P* < 0.0001) and BSHXR could restore it (*P* < 0.01 or *P* < 0.05) (Fig. 5d). TIMP2 protein level was much higher in the model group than that in the control group (*P* < 0.05) and it could be inhibited by BSHXR and HXR treatment (*P* < 0.01 or *P* < 0.05) (Fig. 5e). FGF2, which can be modulated by COX2-PGE2 signaling and MMP, was also hampered in the model group (*P* < 0.05). BSHXR-L and BSHXR-M remarkably improved FGF2 levels (*P* < 0.01) (Fig. 5f). On the other hand, quantification of MMP2 mRNA showed no significant differences among all groups (Fig. 5g). However, we found an elevation of TIMP2 mRNA in the model group (*P* < 0.001), which could be minimized after treatment (*P* < 0.01 or *P* < 0.05) (Fig. 5h). Moreover, the expression pattern of FGF2 mRNA was consistent with that of the FGF2 protein (*P* < 0.001 or *P* < 0.01) (Fig. 5i).

## Discussion

A successful pregnancy calls for well-tuned synchronization between the embryo and the receptive endometrium, which depends on the spatiotemporal molecular mechanisms during implantation [24]. After ovulation, the fertilized embryo reaches the uterine cavity, undergoing positioning, adhesion, invasion, and is finally implanted [7]. Estrogen and progesterone are important hormones regulating implantation and the pregnancy maintenance. Progesterone mainly stimulates stromal decidualization for a receptive endometrium, while a spike of estrogen initiates the embryo implantation [7]. Coordinating functions between progesterone and estrogen ensure an appropriate endometrial receptivity [25]. Whereas, the supraphysiologic levels of steroid hormones during COH were accompanied by significant changes in the endometrial gene- and protein-expression patterns [26, 27]. These abnormalities are associated with poor placentation and adverse obstetric outcomes, such as preeclampsia, small for gestational age, and low birth weight [28–30]. Accordingly, it is very possible that the extremely high progesterone level in COH mice during the peri-implantation period influenced endometrial receptivity and led to pregnancy loss.

Angiogenesis provides a favorable environment required for embryo implantation [31]. In clinical practice, scanning endometrial morphological parameters, such as blood flow, is the current and preferred approach for assessing endometrial function [32]. Increased endometrial vascular permeability is a discernible mark of embryo attachment, which results in the continuation and progressive increase in angiogenesis [33, 34]. Insufficient angiogenesis may result in poor endometrial receptivity [35]. Steroid hormones mediate the spatiotemporal expression of proangiogenic factors during early pregnancy; estrogen mainly induces endometrial vascular permeability, whereas progesterone promotes angiogenesis [36]. These actions are mediated between the steroid hormones and the hormone receptors [8]. ER is expressed in the endometrium in two distinct forms, namely, ERα and ERβ. While ERα is more important for embryo implantation. ERα knockout mice show endometrial hypoplasia and sterility, whereas mice with ERβ deficiency manifest as normal fertility [37, 38]. Progesterone alleviates the effect of estrogen by downregulating the expression of ERα [39]. In another way, ER further modulates the transcription of PR, demonstrating the coordinating functions as well as the antagonism between estrogen and progesterone [7]. Thus, the supraphysiological levels of progesterone might affect ERα and PR expression, leading to endometrial dysplasia and poor endometrial angiogenesis, which was verified in our COH model.

As the results of CD31 immunostaining showed poor vascular development in COH mice, we next explored the expression of angiogenic factors in the endometrium. It is well known that angiogenesis is critically modulated by the oxygen levels in the tissues. A hypoxic microenvironment is necessary for successful implantation [40]. HIF1α is highly expressed in the endometrium and its depletion results in subfertility [41, 42]. VEGF, the downstream factor of HIF1α, is the earliest factor to be detected in conjunction with the angiopoietin system during implantation [43]. As a predominant isoform in the endometrium, VEGFA promotes the maturation of blood vessels and maintains vessel leakiness, whereas ANGPT2 stimulates vessel destabilization to facilitate further sprouting [43]. Our study revealed that COH could disrupt the hypoxic microenvironment via the expression of HIF1α; there was no correlation of COH with VEGFA or ANGPT2. These results indicated a broader role of HIF1α during early pregnancy.

COX2 also plays a critical role in vascular permeability and angiogenesis. Its deficiency leads to implantation failure, which is characterized by reduced angiogenesis [44]. Prostaglandins are chemicals generated from arachidonic acid and COX2 is a key enzyme that mediates the synthesis of prostaglandins [45]. PGE2 is a predominant prostaglandin that distinctly stimulates endometrial vascular permeability in rats [33]. PGE2 acts via binding to various G-protein coupled receptors, and the EP2 receptor is mainly involved in angiogenesis. As shown in this study, the distinct positioning patterns between the COX2 and EP2 receptor suggested that PGE2 produced by the epithelial cells might communicate with the stromal cells via paracrine signaling. Moreover, the endometrial COX2-PGE2 pathway was disturbed in COH mice, which may result from the diminishment of HIF1α. This can be attributed to the fact that the expression of COX2 depends on HIF1α accumulation in endometrial cells [46]. However, the trend of COX2 and EP2 receptor mRNA was inconsistent with HIF1α in this study. Hence, whether and how HIF1α regulates COX2 in the epithelium during implantation needs to be further elucidated.

COH has been attributed to the disturbance in the expression of several genes involved in the degradation of the extracellular matrix [47, 48]. Matrix remodeling is indispensable in angiogenesis and during trophoblast invasion. MMP and TIMP participate in achieving a balance between the promotion and suppression of matrix degradation [24]. MMP2 mRNA is observed in the stroma, whereas MMP9 mRNA is mainly expressed in giant trophoblast cells [49]. Thus, the expression of the MMP9 gene was almost undetectable in the endometrial tissue collected in our study. Both MMP and TIMP can be modulated by PGE2 in early pregnancy [50]. Another pro-angiogenesis factor, FGF2, is known to be induced by the stimulation of the EP2 receptor or MMP and inhibited by COX2 inhibitors, suggesting an upstream modulation of COX2-PGE2 signaling towards FGF2 [51, 52]. Our data revealed that COH could diminish the process of matrix degradation and angiogenesis, possibly through disruption of the COX2-PGE2 pathway.

**Fig. 6:**
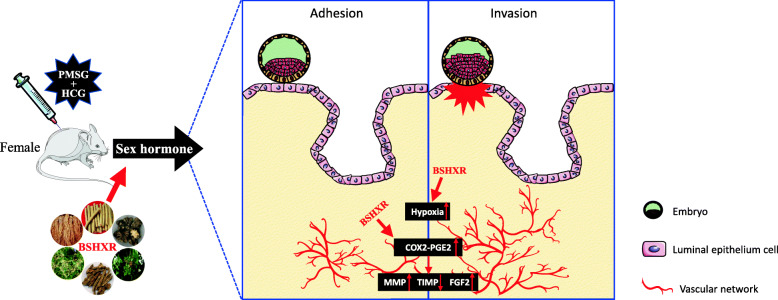
The summary of the experiment. BSHXR alleviates the negative effects of supraphysiological levels of sex hormones caused by COH, and facilitates the embryo implantation by improving endometrial angiogenesis through the COX2-PGE2 pathway and the relevant angiogenic factors

Bushen Huoxue is a predominant TCM principle used for alleviating complications during pregnancy. In the present study, we demonstrated that BSHXR could increase the pregnancy rate and implantation sites in COH mice (Fig. 6). There are two possible causes for these results. First, BSHXR increased the number of ovulations, thereby leading to a higher chance of implantation. The second possibility is that BSHXR directly facilitated embryo implantation. Based on the results of our previous study, there were no notable differences in the implantation sites on ED5 [22, 23]. However, along with embryo development, the rate of fetal loss was higher in COH mice, and could be reversed by treatment using BSHXR [22, 23]. Therefore, it can be interpreted that BSHXR improved the endometrial receptivity and prevented fetal loss. In this aspect, we herein found that BSHXR, especially BSHXR-M, could improve the endometrial weight ratio in mice subjected to COH, revealing a higher level of endometrial development.

Moreover, our study also indicated that the promotion of endometrial development by BSHXR was associated with the restoration of steroid hormones and the corresponding receptors. Since BSHXR could alleviate the abnormally high levels of progesterone, the question arises if BSHXR inhibited ovarian function. In this study, the levels of sex hormones in the control + BSHXR group were similar to those in the control group, demonstrating that BSHXR did not inhibit ovarian function. Moreover, the pregnancy outcome in the control + BSHXR group was comparable to that in the control group, demonstrating the safety of BSHXR administration during pregnancy. Apart from these findings, we further found that BSHXR could increase the expression of angiogenesis factors, such as HIF1α, COX2, PGE2, EP2 receptor, MMP2, MMP9, and FGF2, and also inhibit TIMP2 in the endometrium, all of which could participate in the vascular development during early pregnancy. Intriguingly, BSHXR-M, which equals the commonly used dosage in a clinical setting, was found to be more efficacious than BSHXR-L, BSHXR-H, BSR, and HXR. This result indicates that the medium dose of BSHXR is more suitable for pregnant women. Like many other drugs, there likely exists a certain dose - effect relationship. The efficacy of BSHXR-H is to some extent inferior to BSHXR-M. Furthermore, TCM decoctions that have high drug concentrations tend to be sticky and viscous. On one hand, the absorption of BSHXR-H might have been compromised compared to BSHXR-M. On the other hand, owing to the susceptibility to sensory stimulation, the extremely bitter taste might have disturbed the overall condition of the mice in the BSHXR-H group.

Lastly, BSR and HXR have synergistic and their own selective effects. For example, neither BSR nor HXR could facilitate ERα or PR expression, possibly resulting from the high levels of sex hormones in the BSR and HXR groups. Both BSR and HXR could promote vascular development and HIF1α expression level in mice subjected to COH, although at levels lower than those observed using BSHXR. These results indicated the common effects of BSR and HXR. Additionally, BSR could increase the endometrial PGE2 level more than the increase induced by HXR, while HXR tended to enhance tissue remodeling via MMP2 and TIMP2 compared to that observed using BSR. Collectively, these results suggested that BSR and HXR might have individual and selective effects during angiogenesis. More importantly, separated recipes presented complementary advantages. On ED8, the number of embryos in the BSR group was significantly higher than that in the model group. In contrast, the number of embryos was relatively low in the HXR group. Interestingly, estrogen levels did not change in the control, model, and BSHXR groups, while it increased in the BSR and HXR groups during the peri-implantation period. The combination of BSR and HXR enhanced the efficacy of BSHXR, implying the superiority of the compound prescription.

Our study has some limitations. First, the specific target of BSHXR was not identified. It was unclear how BSHXR improved endometrial receptivity by reducing high levels of hormones. The bidirectional regulatory role of BSHXR on endocrine levels was unknown and it was unclear if BSHXR directly affected stromal cell decidualization. The exact function of individual components in the TCM recipe was also unclear. These factors need to be addressed in future studies. Secondly, given that this study only referred to the effect of BSHXR in endometrial angiogenesis, the significance of BSR and HXR in other aspects of pregnancy should also be evaluated. Lastly, owing to the differences between the reproductive physiology in mice and humans, the mechanism underlying the loss of murine pregnancy may not be completely similar to that observed in human pregnancy.

## Conclusions

Taken together, our study revealed that ovarian hyperstimulation led to a loss of pregnancy and was accompanied by supraphysiological levels of steroid hormones and compromised endometrial angiogenesis. BSHXR could improve pregnancy outcomes by regulating high levels of steroid hormones and promoting endometrial vascularization, thus increasing endometrial receptivity and promoting embryo implantation. BSHXR treatment did not show any side effects during normal pregnancy, and BSR and HXR were found to have their own selective and synergistic effects regarding their beneficial effects in pregnancy. The novel findings in this study will provide a scientific basis for the clinical use of BSHXR for infertile patients during the peri-implantation period in IVF-ET cycles, likely increasing the success rate of embryo implantation.

## Supplementary Information

**Additional file 1.**

**Additional file 2.**

**Additional file 3.**

**Additional file 4.**

**Additional file 5.**

**Additional file 6.**

## Data Availability

The datasets used and/or analyzed during the current study are available from the corresponding author on reasonable request.

## Abbreviations

IVF-ET: In vitro fertilization and embryo transfer
COH: Controlled ovarian hyperstimulation
ED: Embryo day
FET: Frozen embryo transfer
TCM: Traditional Chinese medicine
BSHXR: Bushen huoxue recipe
BSR: Bushen recipe
HXR: Huoxue recipe
PMSG: Pregnant mare serum gonadotrophin
HCG: Human chorionic gonadotropin
ELISA: Enzyme-linked immune sorbent assay
PCR: Polymerase chain reaction
ERα: Estrogen receptor α
PR: Progesterone receptor
HIF: Hypoxia inducible factor
VEGF: Vascular endothelial growth factor
ANGPT2: Angiopoietin 2
COX2: Cyclooxygenase 2
PGE2: Prostaglandin E 2
EP2 receptor: Prostaglandin E receptor 2
MMP: Matrix metallopeptidase
TIMP: Tissue inhibitor of metalloproteinase
FGF2: Fibroblast growth factor 2
