# Supplementary figures and images for: Bushen Huoxue recipe attenuates early pregnancy loss via activating endometrial COX2-PGE2 angiogenic signaling in mice
Source: BMC Complement Med Ther. 2021 Jan 14;21:36. doi: 10.1186/s12906-021-03201-9 (PMC7809844; doi:10.1186/s12906-021-03201-9)

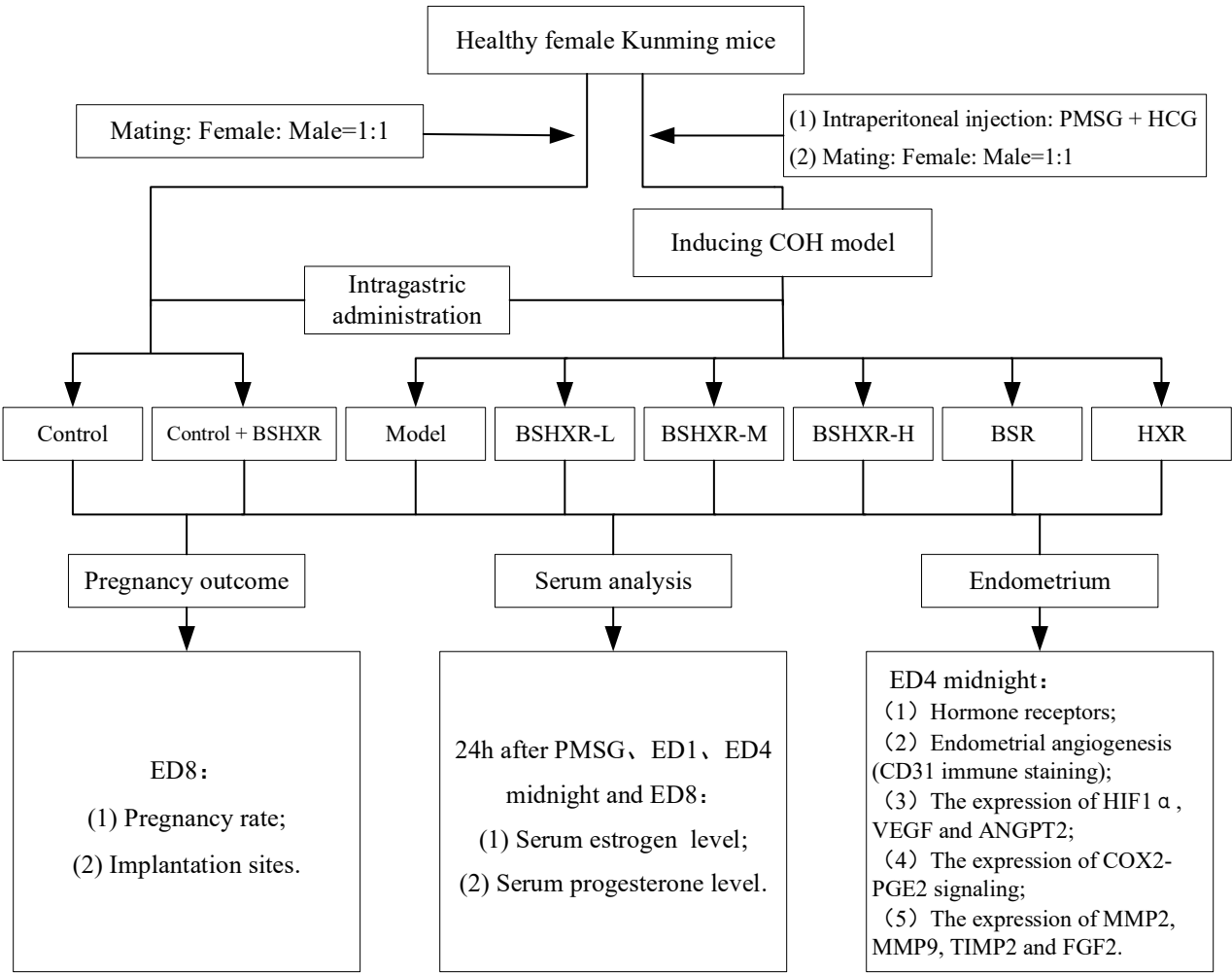

Supplement: Supplementary file 2 — Additional file 2. [file 12906_2021_3201_MOESM2_ESM.pdf]

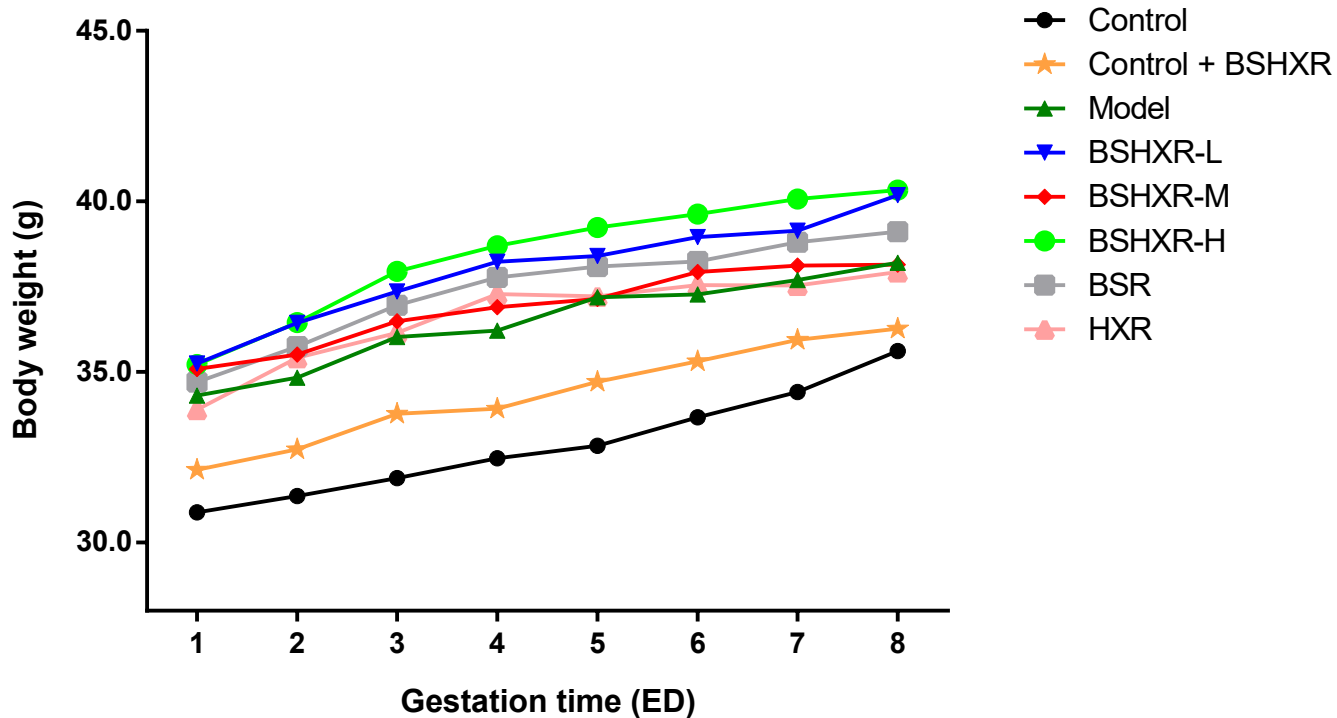

Supplement: Supplementary file 4 — Additional file 4. [file 12906_2021_3201_MOESM4_ESM.pdf]

ER

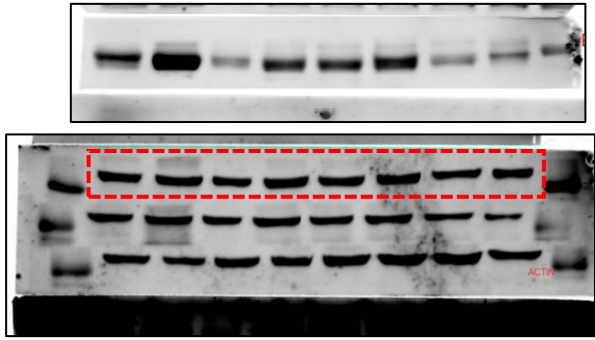

PR

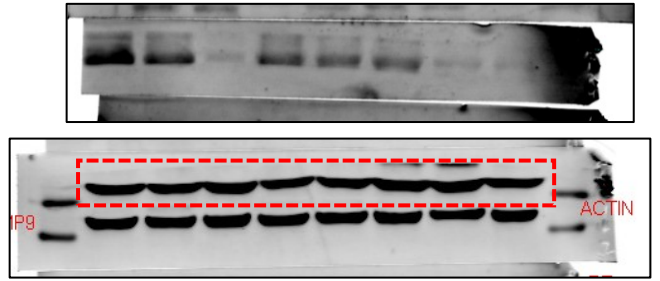

HIF1 $\alpha$

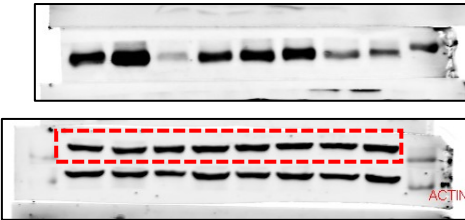

VEGF

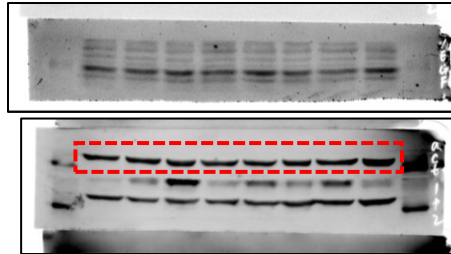

ANGPT2

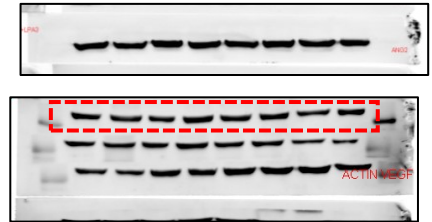

COX2

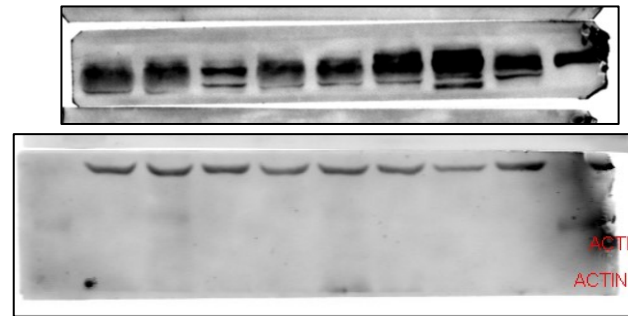

EP2 receptor

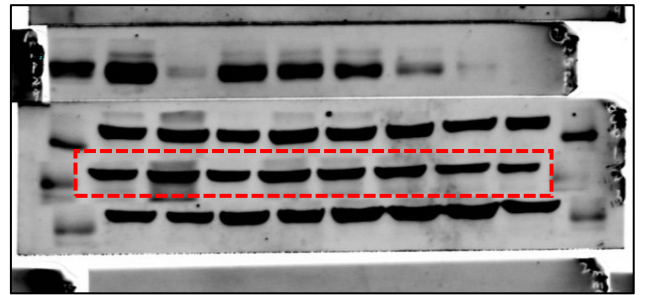

MMP2

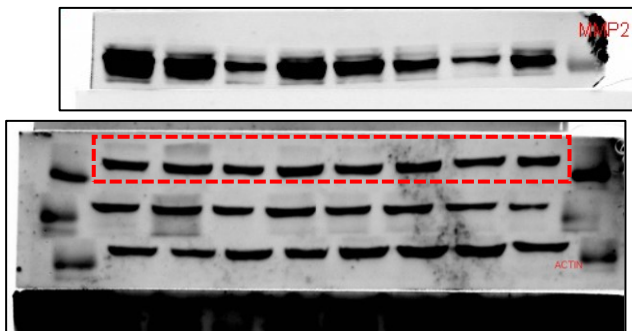

MMP9

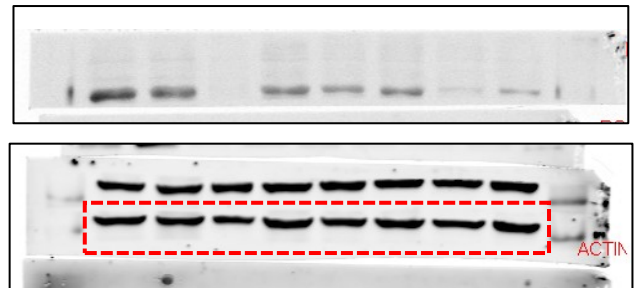

TIMP2

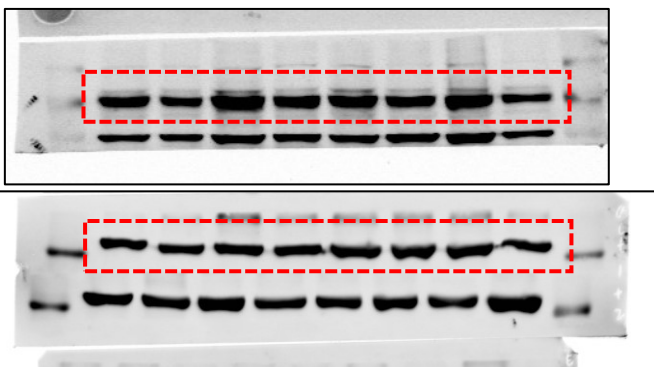

FGF2

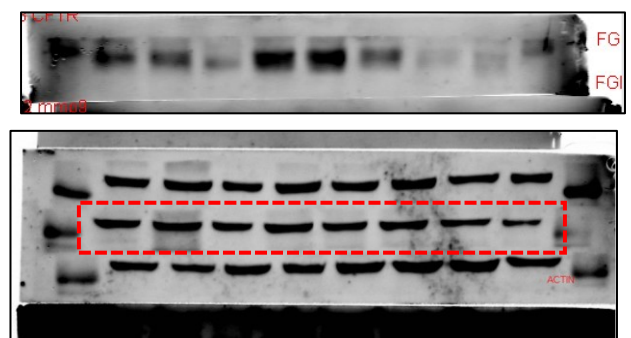

Supplement: Supplementary file 5 — Additional file 5. [file 12906_2021_3201_MOESM5_ESM.pdf]

A

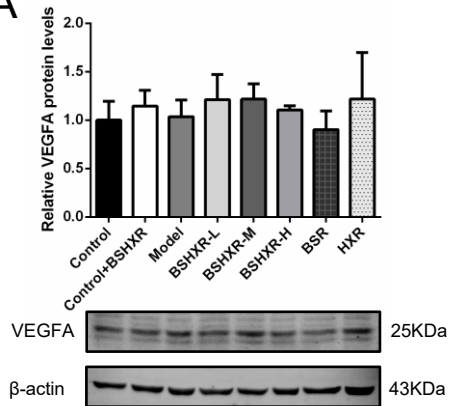

B

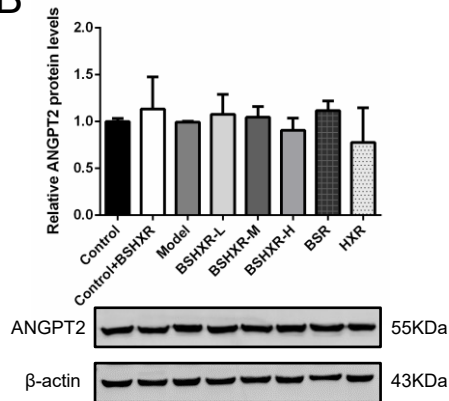

Supplement: Supplementary file 6 — Additional file 6. [file 12906_2021_3201_MOESM6_ESM.pdf]
