# Supplementary material for: Bushen Huoxue recipe attenuates early pregnancy loss via activating endometrial COX2-PGE2 angiogenic signaling in mice
Source: BMC Complement Med Ther. 2021 Jan 14;21:36. doi: 10.1186/s12906-021-03201-9 (PMC7809844; doi:10.1186/s12906-021-03201-9)
Supplement: Supplementary file 3 — Additional file 3. [file 12906_2021_3201_MOESM3_ESM.docx]

**TABLE S1** Specific primer sequences.

| Gene name | Forward (5’ 3’) | Reverse (5’ 3’) |
| --- | --- | --- |
| Ptgs2 | CATCCCCTTCCTGCGAAGTT | GGCCCTGGTGTAGTAGGAGA |
| Ptger2 | ACATGGTGCTTTATCCGGCA | TACTGCCAGACAATCCGCAG |
| Mmp2 | AAAAAGTTGGCATGGAGGCG | GGGCAGCCATAGAAGGTGTT |
| Mmp9 | TACTGGGCGTTAGGGACAGA | TAACGCACAGACCCCCTCTA |
| Timp2 | TTCTTGACATCGAGGACCCG | AAATAGGGGAGGGGGCAGAA |
| Fgf2 | GGCTGCTGGCTTCTAAGTGT | GTCCCGTTTTGGATCCGAGT |
| β-actin | GTTGGAGCAAACATCCCCCA | ACGCGACCATCCTCCTCTTA |
